# The Secretome of Bullous Pemphigoid IgG‐Treated Keratinocytes Induces a Pro‐Inflammatory Eosinophil Response

**DOI:** 10.1111/exd.70265

**Published:** 2026-05-08

**Authors:** Adrian P. Mansini, Lei Bao, Krishan D. Chhiba, Jing Li, Yulu Wang, Haley Gainer, M. Allen McAlexander, Christopher McCrae, Christopher D. Nazaroff, Fei Li Kuang, Kyle T. Amber

**Affiliations:** ^1^ Department of Dermatology Rush University Medical Center Chicago Illinois USA; ^2^ Division of Allergy and Immunology Northwestern University Feinberg School of Medicine Chicago Illinois USA; ^3^ Translational Science and Experimental Medicine, Early Respiratory & Immunology AstraZeneca Gaithersburg Maryland USA; ^4^ Translational Science Johnson and Johnson Innovative Medicine Spring House Pennsylvania USA; ^5^ Department of Ophthalmology Rush University Medical Center Chicago Illinois USA

**Keywords:** allergy, autoimmune blistering disease, eosinophil, epithelial immunity, pemphigoid

## Abstract

Bullous pemphigoid (BP) is an autoimmune blistering disease whereby the cutaneous antigens BP180 and BP230 are targeted by autoantibodies. Skin lesions in BP are characterized by an abundance of eosinophils. We recently demonstrated that the treatment of keratinocytes with antibodies from patients with BP induces a robust inflammatory response with release of numerous cytokines, chemokines, complement factors, and proteases. We thus questioned whether this keratinocyte inflammatory response was capable of directly inducing an inflammatory response in eosinophils. We therefore treated human eosinophils with conditioned media from keratinocytes treated with IgG from patients with BP (BP‐IgG), or healthy controls (control‐IgG) with or without supplemental IL‐5. Flow cytometry revealed upregulation of CD107a/CD107b, markers of degranulation, on eosinophils treated with supernatants from BP‐IgG relative to control‐IgG treated keratinocytes. A decrease in CCR3 and CD101 was also identified. Functional activity of eosinophils was confirmed by performing a multiplex immunoassay on eosinophil supernatants. This revealed significant upregulation IL‐6, IL‐8, LIF, TGFα, MCP‐4, MMP‐9, and MMP‐10. Supplemental IL‐5 did not appear to significantly influence these responses. Our data demonstrate that the inflammatory activation of keratinocytes by BP‐IgG affects eosinophils, driving phenotypic changes consistent with degranulation, as well as a pro‐inflammatory and proteolytic response.

## 1 Background

Bullous pemphigoid (BP) is an autoimmune blistering disease characterized by the presence of autoantibodies against the cutaneous basement membrane zone antigens BP180 and/or BP230, and by an abundance of activated tissue eosinophils [1, 2]. Despite this abundance of eosinophils, their pathogenicity in BP remains unclear. In a murine adoptive transfer model of BP180‐specific CD4+ cells, subepidermal separation with upregulation of IL‐5 and eosinophil infiltration occurred. Treatment with an anti‐IL‐5 antibody significantly reduced this disease severity [3]. Likewise, the IL‐5 levels have correlated with affected body surface area [4]. Yet, in a clinical trial of a mepolizumab, a monoclonal antibody targeting IL‐5, treatment failed to significantly decrease disease severity or ablate tissue eosinophils [5]. This raises the question as to whether a skin‐eosinophil axis exists that propagates local disease.

We recently demonstrated that affinity‐purified antibodies from patients with BP induce a broad inflammatory response by keratinocytes, including cytokines, chemokines, complement components and proteases [6]. Given this finding, we questioned whether the keratinocyte inflammatory milieu released as response to IgG from BP patients (BP‐IgG) was sufficient to activate eosinophils an establish a skin‐eosinophils axis.

### 1.1 Questions Addressed

Is the keratinocyte inflammatory response induced by IgG from BP patients sufficient to induce an activated phenotype in eosinophils?

## 2 Experimental Design

### 2.1 Patients

The study was approved by the Institutional Review Board (IRB#20121406). The study was performed in accordance with the Declaration of Helsinki. After written informed consent, blood was drawn from patients with a confirmed diagnosis of BP based on current international guidelines [7]. This included (1) clinical suspicion for BP, (2) positive direct immunofluorescence demonstrating deposition of IgG along the cutaneous basement membrane zone, and (3) a positive serologic test detecting BP180 and/or BP230 antibodies, and/or indirect immunofluorescence demonstrating deposition along the roof of salt‐split skin. All patients selected had detectable BP180 antibodies. Serum samples were stored at −80C until ready for use.

### 2.2 Affinity Purification

Affinity purification was performed on serum samples utilizing the Nab Protein G spin Column (Thermo Fischer Scientific, Waltham, MA). Antibodies were pooled in equal parts from four patients to minimize variability in the ratio of BP180/BP230 antibodies. Purified IgG was subsequently washed using Amicon Ultra‐15 centrifugal filter units with a 3 kDa filter in PBS (MilliporeSigma, Burlington, MA), followed by azide removal (Nanopartz, Loveland, CO). Purified IgG was assessed for endotoxin contamination by Limulus Amebocyte lysate test and was consistently negative. For control IgG, each biologic replicate utilized pooled IgG from different vendors to increase diversity of the normal control (MP Biochemicals, Irvine, CA; Cell Sciences, Newburyport, MA; Sigma‐Aldrich, St. Louis, MO).

### 2.3 Human Eosinophil Purification

Blood was collected from healthy donors in K2EDTA coated tubes. 60 mL of blood was collected per donor. Eosinophils were subsequently purified using the MACSexpress whole blood isolation kit (Miltenyi Biosciences, Gaithersburg, MD). Initial purity was assessed by flow cytometry with subsequent confirmations performed by placing a smear or isolated cells on a slide and staining with Giemsa. Cell viability was confirmed by Trypan blue. Purity and viability were both confirmed at above 90% and 95%, respectively. Purified eosinophils were resuspended in RPMI with 10% heat inactivated FBS (Thermo Fisher Scientific, Waltham, MA).

### 2.4 Primary Human Keratinocyte Culture

Primary adult human keratinocytes (Thermo Fischer Scientific, Waltham, MA) were cultured in EpiLife Medium with Human Keratinocyte Growth Supplement in a CO_2_ incubator and grown on 60 mm dishes to approximately 70% confluence. Cells were subsequently treated with 2 mg/mL of purified BP‐IgG or control‐IgG overnight. The conditioned supernatant was subsequently removed and stored at −80°C until use.

### 2.5 Eosinophil Treatment With Conditioned Supernatants

Eosinophils were plated at a concentration of 4 × 10^6^/mL for treatment with supernatants for flow cytometry and Luminex assays. An equal volume of conditioned keratinocyte supernatant was subsequently added to the eosinophils cultured in RPMI with 10% FBS and incubated for 4 or 6 h for flow cytometry and Luminex assays, respectively. To account for potential eosinophil FcR stimulation from IgG itself, we additionally utilized a keratinocyte‐free control supernatant spiked with IgG. Before adding keratinocyte supernatants, some eosinophils were pre‐stimulated with 1 ng/mL of IL‐5 (Biolegend, San Diego, CA), a dose chosen to reflect priming of eosinophils without complete activation [8], or 5 ng/mL for longer‐term incubation for Luminex assays. After 4 h, cells were processed for flow cytometry. In a separate experiment with distinct donors, supernatants were extracted for a Luminex assay. As an additional control, we utilized control‐IgG spiked into Epilife with Human Keratinocyte Growth Supplement without keratinocytes.

### 2.6 Flow Cytometry and Multiplex Immunoassay

Flow cytometry and multiplex immunoassay protocols are described in the Data S1 section.

### 2.7 Data Analysis and Statistics

To compare median fluorescent intensity of surface markers, we utilized Friedman's test with uncorrected Dunn‐test based on a priori comparisons of BP‐IgG versus Control‐IgG supernatant treated eosinophils with or without supplemental IL‐5. Cytokine and chemokine concentrations were found to follow a non‐normal distribution by the Shapiro–Wilk test and were thus analysed by multiple Mann–Whitney testing. Pairwise comparison of BP‐IgG vs. Control‐IgG keratinocyte supernatant was performed with or without IL‐5. Data were analysed using Prism 10.2.1 software (Graphpad, La Jolla, CA). All tests were 2 tailed with significance defined as *p* < 0.05.

## 3 Results

### 3.1 Treatment of Eosinophils With Conditioned Supernatants From BP‐IgG Treated Keratinocytes Increases Expression of Granule‐Associated Cytotoxic Proteins

**FIGURE 1:**
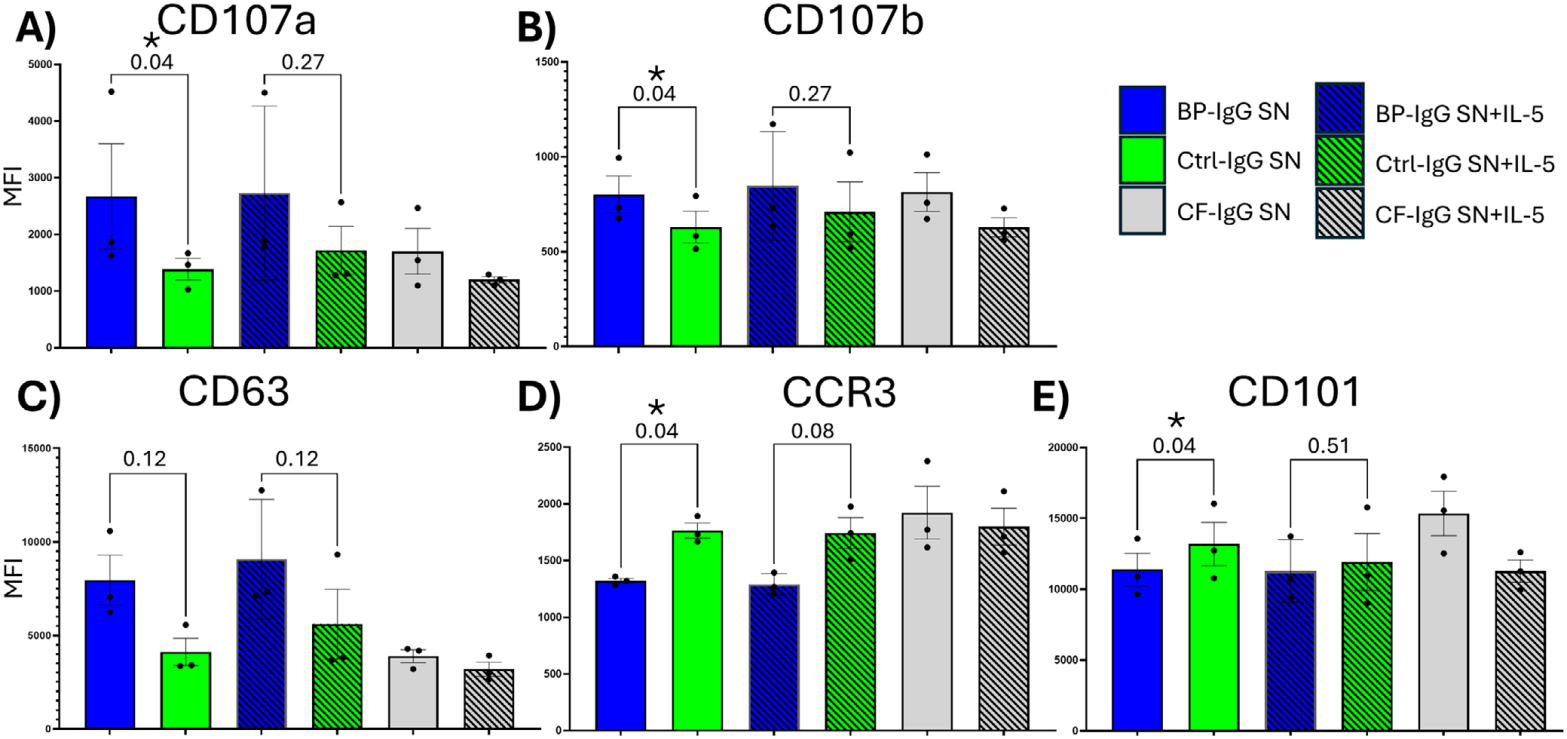
Flow cytometry of eosinophils treated with supernatants from BP‐IgG or Control‐IgG treated primary keratinocytes. Bar plots demonstrate significant upregulation of (A) CD107a, (B) CD107b and (C) a trend towards increased CD63 on eosinophils treated with BP‐IgG treated keratinocyte supernatants relative to control‐IgG treated keratinocyte supernatants. (D) CCR3, and (E) CD101 are decreased on eosinophils treated with BP‐IgG treated keratinocyte supernatants. Data shown is from eosinophils purified (with or without 1 nM IL‐5 priming) from healthy donors (*n* = 3), treated for 4 h with keratinocyte supernatants generated by treating primary keratinocytes with pooled BP‐IgG (*n* = 4), or control IgG (*n* = 3) overnight. IgG spiked into keratinocyte media in the absence of keratinocytes (CF, cell free) was used as an additional negative control. Friedman test with uncorrected Dunn's‐test. Data shown is median fluorescence intensity shown as mean ± SEM. CF—Cell free, MFI—Median fluorescence intensity SN—Supernatants.

Flow cytometry revealed upregulation of the degranulation markers CD107a/CD107b (Figure 1A,B) on eosinophils treated with supernatants from BP‐IgG treated keratinocytes. This was only significant in the absence of IL‐5 priming. CD63, another marker of eosinophil degranulation demonstrated a similar trend, but did not reach statistical significance (Figure 1C). Interestingly, we identified a decrease in CCR3 on eosinophils treated with supernatants from BP‐IgG keratinocytes, a finding associated with tissue eosinophils (Figure 1D) [4]. CD101, was surprisingly decreased on eosinophils treated with BP‐IgG‐stimulated keratinocytes, a finding more consistent with anti‐inflammatory eosinophils [9] (Figure 1E). Other markers, including CD62L, CD69, CD66b, CXCR4, CD11b, and CD274 were not significantly affected (not shown).

### 3.2 Treatment of Eosinophils With Conditioned Supernatants From BP‐IgG Treated Keratinocytes Induces a Broad Inflammatory Response

Next, we sought to confirm functional activation of eosinophils, by comparing cytokine, chemokine, and metalloprotease response in eosinophils treated with either BP‐IgG or control‐IgG treated keratinocyte supernatants. As such, we performed a multiplex immunoassay on eosinophil supernatants. To account for the known inflammatory response of keratinocytes as a response to BP‐IgG, we subtracted keratinocyte supernatant values from stimulated eosinophil values for BP‐IgG or Ctrl‐IgG, respectively.

**FIGURE 2:**
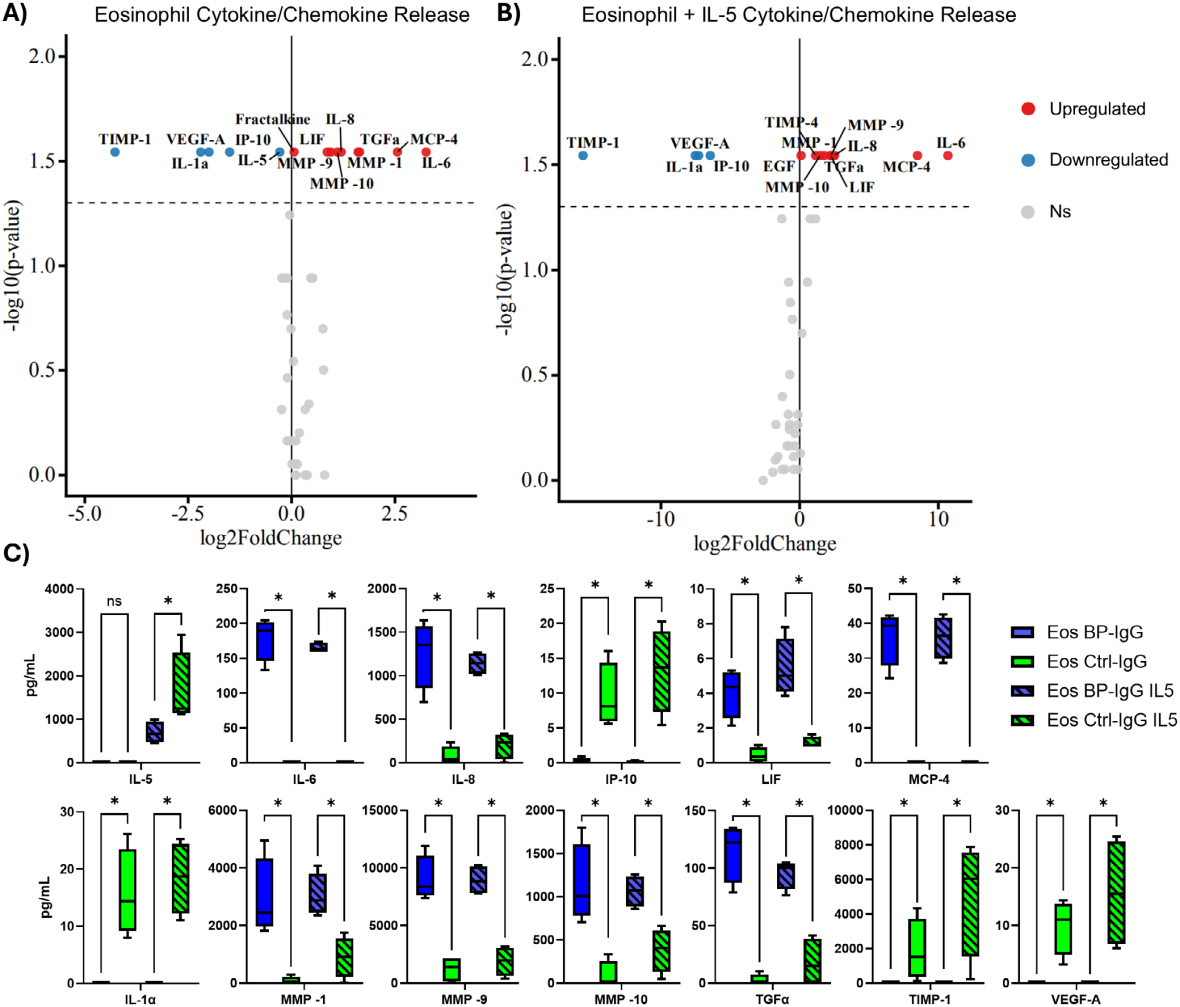
Luminex multiplex immunoassay of eosinophil supernatants after stimulation with BP‐IgG or Control‐IgG treated primary human keratinocyte conditioned supernatants. Volcano plots for pairwise comparisons of (A) eosinophils treated with BP‐IgG vs. Ctrl‐IgG stimulated keratinocyte supernatants without supplemental IL‐5 or (B), with supplemental IL‐5 (5 ng/mL). The dotted line represents the significance cutoff (*p* < 0.05). Mann–Whitney test. (C) Individual cytokines/chemokines differentially expressed between eosinophils treated with BP‐IgG vs. Ctrl‐IgG stimulated keratinocytes with or without IL‐5. Data shown are of eosinophil supernatants from eosinophils purified from healthy donors (*n* = 4) with or without IL‐5, treated with keratinocyte supernatants for 6 h, generated by treating primary keratinocytes with pooled BP‐IgG (*n* = 4), or control IgG (*n* = 3) overnight. Data is normalized to cytokine/chemokine levels in the respective keratinocyte supernatants. Data shown as box and whisker plots indicates media minimum, and maximum with pairwise comparison either without or with IL‐5 by Mann Whitney test. *p < 0.05.

Pairwise comparisons of BP‐IgG versus Ctrl‐IgG supernatant treated eosinophils demonstrated upregulation of IL‐6, IL‐8, LIF, TGFα, MCP‐4, MMP‐9, and MMP‐10. IL‐1α, IP‐10, TIMP‐1, and VEGF‐A were conversely decreased. Supplemental IL‐5 (5 ng/mL) did not significantly impact the results (Figure 2A–C). Interestingly, IL‐5 was relatively decreased in eosinophils treated with BP‐IgG supernatants, though we hypothesize that this is due to eosinophil consumption of IL‐5 in the setting of higher activation [10].

## 4 Conclusions and Perspectives

Our data demonstrates that the inflammatory activation of keratinocytes by BP‐IgG affects eosinophils, driving phenotypic changes consistent with degranulation, as well as a pro‐inflammatory and proteolytic response. Upregulation of MMP‐9 is particularly relevant, as granulocyte derived MMP‐9 has previously been demonstrated to be a key factor in cleaving BP180, although there remains some controversy whether MMP‐9 alone is sufficient to cleave BP180 [11, 12]. TGF‐α has also recently been shown to regulate BP pathogenesis by downregulating BP180 expression, upregulating metalloproteinases, and enhancing keratinocyte chemokine secretion [13]. TGF‐α levels notably correlated with disease severity. MCP‐4 is also of note, as this chemokine is capable of driving further eosinophil activation and inflammation [14].

Interestingly, our previous work treating keratinocytes with BP‐IgG did not demonstrate prototypical drivers of eosinophil chemotaxis or activation such as eotaxin or IL‐5 [6]. However, several inflammatory molecules upregulated by keratinocytes such as CXCL16, TGF‐β1, and IL‐8 can affect eosinophil function. We additionally only assessed a large panel of inflammatory proteins and transcriptomics. Thus, proteins not assessed, or non‐protein inflammatory mediators could exist. Upregulation of keratinocyte *PLA2G4A* suggests an increase in prostaglandin and leukotriene production which can also drive eosinophil function. Thus, although our findings demonstrate that the inflammatory milieu released by keratinocytes in response to BP‐IgG induces an inflammatory eosinophil response, future work will be needed to determine the primary contributors. Likewise, measurement at multiple time courses would provide further insight into activation and degranulation kinetics.

Our results surprisingly did not demonstrate a significant difference in response based on IL‐5 priming. This can suggest IL‐5 independent piecemeal degranulation programs or methodological limitations such as dosing parameters which affect the degree of activation. We notably used lower doses (1 ng/mL or 5 ng/mL) of IL‐5 rather than the standard 10 ng/mL to avoid introducing experimental noise that would mask the effect of keratinocyte derived inflammatory markers relative to the highly potent effect of IL‐5.

A significant limitation of our study is the use of a simplified model with keratinocytes and eosinophils in isolation. While this allowed us to demonstrate a direct impact of the keratinocyte secretome on eosinophils, this simplified model excludes cytokines from other cellular sources and the cutaneous microbiome. Yet, we hypothesize that these would only exacerbate the eosinophil inflammatory response. For example, epicutaneous exposure to *Staphylococcus aureus* , has been shown to increase eosinophil recruitment [15]. TSST‐1+ *Staphylococcus aureus* colonization has previously been demonstrated in patients with BP [16]. We found that MyD88 regulates a large portion of the keratinocyte inflammatory response to BP‐IgG [6]. Thus, TLR2 stimulation of keratinocytes, which utilizes MyD88 as an adaptor protein would presumably exacerbate the BP‐IgG induced keratinocyte inflammatory response. Likewise, we have recently demonstrated activation of lesional eosinophils in BP with expression of HLA‐DQ and CD86 [17]. Thus, contributions beyond keratinocyte‐eosinophil cross talk are necessary to fully recapitulate the eosinophil response in BP. Additionally the use of control‐IgG treated keratinocytes does not allow determination of FcR‐dependent and ‐independent effects. This simplified model does, however, allow us to determine that the inflammatory milieu from BP‐IgG keratinocytes is at least contributory towards the eosinophil inflammatory response.

Additionally, our study demonstrated phenotypic changes of surface markers consistent with degranulation (CD107a/CD107b), but not activation (CD62L, CD69, CD66b). This can occur for several reasons, including pre‐activation of eosinophils, piecemeal degranulation [18], differential kinetics of degranulation versus activation surface markers, and non‐canonical activation pathways such as stimulation with leukotrienes, complement fragments, or protease signalling. The significant upregulation of MMP9 by eosinophils is consistent with their activation [19]. Likewise, IL‐5 levels were significantly decreased in eosinophils treated with BP‐IgG keratinocyte supernatants with supplemental IL‐5 in contrast to other activation markers such as IL‐6 and IL‐8. This reduction in IL‐5 is presumably due to utilization of IL‐5 during the activation process [5]. Nonetheless, despite the complex mechanism of eosinophil degranulation, our study demonstrates a pro‐inflammatory eosinophil response.

While we demonstrate that the keratinocyte secretome as a response to BP‐IgG is capable of activating eosinophils and inducing upregulation of inflammatory mediators and MMP9, the pathogenicity of eosinophils in BP overall remains unclear. Likewise, the specificity to BP remains unclear. We have, however, demonstrated distinct keratinocyte inflammatory reactions upon treatment with BP‐IgG versus that from patients with laminin‐332 pemphigoid [20], suggesting that targeting of different antigens does result in distinct inflammatory responses.

While eosinophil granule proteins themselves have been shown to be pathogenic to keratinocytes [21, 22] as well as ex vivo studies demonstrating pathogenic effects of activated eosinophils in a model of BP [23], trials assessing eosinophil depletion as a therapeutic strategy in BP have been unsuccessful (NCT04612790, NCT01705795). As noted previously, however, mepolizumab only ablated circulating eosinophil populations, but not lesional [5]. Thus, it is unclear how activated eosinophils contribute to disease pathogenesis. Nevertheless, the abundance of tissue eosinophils and frequency of peripheral eosinophilia in BP suggests some role in disease pathogenesis, even if not the main driver. It is possible eosinophils play a role in restoring homeostasis such as in wound repair or tissue remodelling. Together, these data identify the keratinocyte secretome as a previously unrecognized driver of eosinophil degranulation in BP, positioning epithelial cells not merely as passive targets, but as active amplifiers of autoantibody‐mediated inflammation. Thus, our findings suggest that targeting keratinocyte‐derived mediators may represent a more upstream approach that would still affect eosinophil responses.

## Author Contributions

Conceptualization – K.T.A., M.A.M., CM, CDN; Formal Analysis – K.T.A., K.D.C., F.L.K.; Investigation – A.P.M., LB, K.D.C., HG, JL, YL, F.L.K.; Methodology – K.T.A., F.L.K.; Project administration – K.T.A., Resources – M.A.M., CM, CDN; Supervision – K.T.A., F.L.K.; Visualization – K.D.C., K.T.A.; Writing – original draft – K.T.A.; Writing – review and editing – A.P.M., LB, KDC, HG, JL, YW, M.A.M., CM, CDN, F.L.K., K.T.A.

## Funding

This work was supported by Astra zeneca, 10046533. National Institute of Allergy and Infectious Diseases, T32AI083216‐15, K23AI171085.

## Ethics Statement

The study was approved by the Institutional Review Board (IRB#20121406) on 2/22/2021. The study was performed in accordance with the Declaration of Helsinki.

## Consent

Written informed consent was obtained from subjects.

## Conflicts of Interest

This work was supported in part by Astra Zeneca. AM, CM, and CN are past or present employees of AstraZeneca and may hold stock or stock options.

## Supporting information


**Data S1:** exd70265‐sup‐0001‐Supinfo.docx. **Supporting Informations and Methods:** Methods for flow cytometry and multiplex immunoassays.

## Data Availability

The data that support the findings of this study are available from the corresponding author upon reasonable request.
