# Supplementary material for: The Secretome of Bullous Pemphigoid IgG‐Treated Keratinocytes Induces a Pro‐Inflammatory Eosinophil Response
Source: Exp Dermatol. 2026 May 8;35:e70265. doi: 10.1111/exd.70265 (PMC13154717; doi:10.1111/exd.70265)
Supplement: Supplementary file 1 — Data S1: exd70265‐sup‐0001‐Supinfo.docx. Supporting Informations and Methods: Methods for flow cytometry and multiplex immunoassays. [file EXD-35-e70265-s001.docx]

**Supplementary Materials and Methods:**

***Flow Cytometry:*** For select wells, eosinophils were treated with keratinocyte supernatants and/or IL-5 as described above. As a positive control for eosinophil degranulation/activation, one well of eosinophils from each patient was primed with 50ng/mL of IL-5 for 30 minutes at 37°C followed by stimulation with 100nM of C5a (R&D, Minneapolis, MN) for 30 minutes at 37C (not shown). Following these treatments, eosinophils were incubated with antibody-fluorophore combinations for 30 minutes at room temperature protected from light. The antibody-fluorophore combinations used in our panels included: CD45-Pacific Orange, Siglec-8-PE, IL-5R-PE, Siglec-8-APC, CXCR4-APC, CD107A-FITC, CD107B-FITC, CD69-PE-Cy7, CD11b-PE-Cy7, CD101-AF647, CD274-PerCP-C5.5, CD62L-PerCP-C5.5, CCR3-APC-C7, FcERI-APC-Cy7, CD63-Pacific Blue and CD66b-Pacific Blue. Following antibody staining, plates were centrifuged at 300g for 10 minutes and supernatants were removed. Cells were resuspended in BD 1x Pharm Lyse Buffer followed by three washes with FACS wash buffer (0.1% BSA, 1mM EDTA, 1x PBS). After the final wash, cells were resuspended in FACS staining buffer (1% BSA, 1mM EDTA, 1x PBS) and immediately acquired on a BD FACSymphony A3 cell analyzer. Compensation was performed using single-color controls prepared with compensation beads for each fluorochrome. Eosinophils were identified by gating on high side scatter, CD45-positive, Siglec-8-positive events. Both median fluorescence intensity (MFI) and percent positive were used for analysis of the various event populations.

***Multiplex Immunoassay:*** Multiplex measurements of supernatants were performed using the Luminex 200 System (Luminex, Austin, Texas, USA) by Eve Technologies Corp. (Calgary, Alberta, Canada). Eve Technologies’ Human MMP/TIMP 13-Plex Discovery Assay, and Eve Technologies’ Human Cytokine 96-Plex Discovery Assay were used to measure supernatant cytokine, chemokine, and metalloprotease expression according to the manufacturer’s instructions. To adjust for inflammatory markers found in the keratinocyte supernatants themselves, eosinophil samples were normalized by subtracting the concentration of each analyte in 1:2 dilution of keratinocyte supernatant in RPMI+10% heat inactivated FBS from the respective eosinophil concentrations. Values were found to follow a non-normal distribution by the Shapiro-Wilk test and were thus analyzed by multiple Mann-Whitney testing. Pair wise comparisons of BP-IgG vs Control-IgG keratinocyte supernatant were performed with or without IL-5.
